# Design Optimisation of a Magnetic Field Based Soft Tactile Sensor

**DOI:** 10.3390/s17112539

**Published:** 2017-11-03

**Authors:** Gregory de Boer, Nicholas Raske, Hongbo Wang, Junwai Kow, Ali Alazmani, Mazdak Ghajari, Peter Culmer, Robert Hewson

**Affiliations:** 1 School of Mechanical Engineering, University of Leeds, Woodhouse Lane, Leeds LS2 9JT, UK; el11jwk@leeds.ac.uk (J.K.); a.alazmani@leeds.ac.uk (A.A.); p.r.culmer@leeds.ac.uk (P.C.); 2 Department of Aeronautics, Imperial College London, South Kensington Campus, London SW7 2AZ, UK; n.raske@imperial.ac.uk (N.R.); r.hewson@imperial.ac.uk (R.H.); 3 Center for Micro-BioRobotics, Istituto Italiano di Tecnologia, Viale Rinaldo Piaggio 34, 56025 Pontedera, Italy; hongbo.wang@iit.it; 4 Dyson School of Design Engineering, Imperial College London, 10 Princes Gardens, Kensington Princes Gardens, London SW7 1NA, UK; m.ghajari@imperial.ac.uk

**Keywords:** tactile sensing, sensitivity, optimisation, magnetic fields, force measurement

## Abstract

This paper investigates the design optimisation of a magnetic field based soft tactile sensor, comprised of a magnet and Hall effect module separated by an elastomer. The aim was to minimise sensitivity of the output force with respect to the input magnetic field; this was achieved by varying the geometry and material properties. Finite element simulations determined the magnetic field and structural behaviour under load. Genetic programming produced phenomenological expressions describing these responses. Optimisation studies constrained by a measurable force and stable loading conditions were conducted; these produced Pareto sets of designs from which the optimal sensor characteristics were selected. The optimisation demonstrated a compromise between sensitivity and the measurable force, a fabricated version of the optimised sensor validated the improvements made using this methodology. The approach presented can be applied in general for optimising soft tactile sensor designs over a range of applications and sensing modes.

## 1. Introduction

Tactile sensors enable robots to interact with humans and the environment with a high level of accuracy and enhance their abilities for dexterous manipulations. Concurrently, technical challenges remain for soft tactile sensing systems to reach human-level performance [1]. Various physical transducer mechanisms (e.g., resistive, capacitive, magnetic, piezoelectric, piezoresistive) have been introduced to develop soft tactile sensors [2], with each design exhibiting their own set of advantages and disadvantages. In general, resistive, capacitive, piezoelectric/piezoresistive tactile sensors have high spatial resolution and performance [2,3], which are easy to implement on ultra-thin layers, but they do not measure tri-axis force nor are they deformable. An encapsulation of soft skin on the working surface protects the sensor electronics and wires but this lowers the performance (in terms of hysteresis, sensitivity, and bandwidth) and requires a complex fabrication process [4]. Optical tactile sensors such as TACTIP [5] are durable and have high spatial resolution but are limited by complex computation between inputs and outputs, high power consumption, and are difficult to use to investigate a large sensing area. BioTac [6] is a bio-inspired fingertip which uses a conductive liquid to transfer force to resistance and is capable of force, contact, temperature, and vibration measurements. BioTac has been integrated into a range of robotic hand fingertip devices but remains expensive, complicated, and difficult to use over a large sensing area.

**Figure 1:**
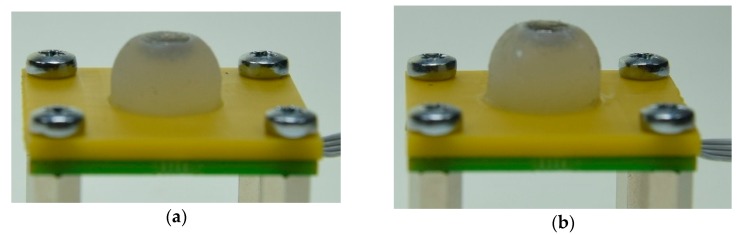
Photographs of the MagOne sensor designs. (**a**) As developed by Wang et al. [9]. (**b**) From the optimisation of sensitivity considered in this study.

Originally presented by Clark [7], magnetic field-based tactile sensors use a remote sensing approach which is inherently durable, deformable, low-cost, easy to fabricate, and easy to integrate with existing robotic systems [8]. Wang et al. [9] introduced a comprehensive design methodology to enable researchers from different disciplines to design high performance soft tactile sensors for specific applications. A single element soft tri-axis tactile sensor, MagOne (Figure 1a), was fabricated as a prototype case study and achieved a force measurement resolution of approximately 1 mN with good repeatability and low hysteresis (3.4%). The performance of MagOne was comparable to commercial rigid force/torque sensors and cost about £10 to fabricate. All aspects, including geometry, fabrication, calibration, performance evaluation, advantages and disadvantages, were investigated to design a high performance soft tactile sensor based on the magnetic field measurement. However, given the complex relationship between magnetic field and force, the MagOne design was not optimised for the multiple design objectives associated with a range of diverse applications. The effect of a range of design variables (e.g., geometry and material properties) on the sensor performance needed to be characterised and from this an optimal design could be determined. Multi-element magnetic field based soft sensors such as Tomo et al. [10] for soft-skin applications, de Oliveria et al. [11] for multimodal sensing and Wang et al. [12] as an extension of MagOne have been recently established. The optimisation principles developed in this work are also applicable to these cases, leading to a means of establishing improvements and optimising the performance of these sensors.

This paper investigates the design optimisation of the MagOne sensor, for this purpose Finite element (FE) simulations were developed to determine the magnetic field and structural behaviour under load. Optimisation studies have been conducted using simulated results of magnetic fields [13,14] and compliant structures/mechanisms [15,16,17]; however, this paper is the first to apply design optimisation techniques in order to simultaneously address these interactions. Studies have also been conducted exploring the optimisation of soft sensors [18,19]. However, the sensors investigated have different operating modes to that of MagOne and the research outcomes did not produce optimised designs, but rather strategies for optimising the output sensing range by control of the input parameters. Genetic programming (GP) was used in this work as a metamodel to produce accurate relationships of the non-linear sensor response over the range of parameters investigated, which is a well-established approach in design optimisation [20]. The optimised designs presented were calculated using a combination of genetic algorithms (GAs) and heuristic solvers based on the relationships generated by GP; such solution procedures have been used previously for this purpose [21].

The motive for the optimisation was to improve the function of the sensor, as defined by its ability to accurately characterise an applied force. For the MagOne device this means a small change in applied load corresponds to a large change in the sensed magnetic field where the relationship between applied force and sensed magnetic field was defined as the sensitivity. The objective of the optimisation study was, therefore, to minimise sensitivity over a range of design variables. A second, and conflicting, objective was introduced to maximise the largest applied load that the sensor can characterise. This ensured that it will have a useful operating range. The design variables were selected to parametrise the geometry and material properties of the sensor, which can be altered as part of the fabrication process. Constraints on the measureable force, given as part of the sensor specification, were imposed and included in the design strategy. Shear loading conditions were investigated in order to optimise the sensor design over a representative range of operational displacements. The optimised designs were subsequently validated by fabricating and experimentally testing the new sensor (Figure 1b), thus demonstrating the accuracy of the computational simulations and the improvements in sensitivity obtained by design optimisation.

The application of this approach to the optimisation of sensitivity subject to a load range (or conversely the optimisation of a sensing range subject to the load range, or a multi-objective optimisation process where both sensitivity and sensing range are considered in combination) can be readily applied to different sensing modalities, such as those based on capacitive based sensing where compression of a soft material and its mechanical response forms the basis of converting the sensor deformation to the resulting force [22]. Indeed, the check for the uniqueness of response and computational electro-mechanical characterisation of the response of a wide range of sensors could potentially be characterised and optimised in terms of geometry [23] so long as a deterministic computational model of the sensor can be derived [24,25,26]. The general method described in this paper, where the sensor is parametrised by design variables which are then optimised based on numerical simulations and metamodels of the input/output response, is applicable to the design of a range of soft tactile sensors.

## 2. Materials and Methods

### 2.1. Sensor Concept

**Figure 2:**
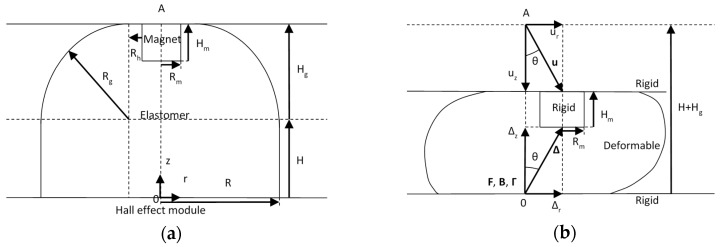
Cross-sectional sketch of the MagOne sensor. (**a**) Unloaded. (**b**) Loaded in both normal and shear directions.

This work is focused on the optimisation of a magnetic sensor concept developed by Wang et al. [9] which comprised of a 3D Hall module, a deformable elastomer body and an embedded magnet as illustrated in Figure 2a. The sensor, known as MagOne, worked by measuring the magnetic field produced at the origin 0, by means of the induced electric field in the Hall effect module. By displacing the magnet from A, the change in magnetic field was identified and then the force applied was determined from knowledge of the mechanical behaviour of the elastomer under load.

Both normal (*z*-axis) and shear (*r*-axis) loading were considered, in which contact and friction occurred between the upper and lower surfaces of the sensor and the rigid surfaces used for indentation. Figure 2b illustrates normal and shear loading of the sensor due to a displacement $u=\left(u_{r},u_{z}\right)$ of the magnet, where $\Delta =\left(\Delta_{r},\Delta_{z}\right)$ is the distance between the magnet and the Hall effect module, θ is the shear angle, $B=\left(B_{r},B_{z}\right)$ is the magnetic field, $F=\left(F_{r},F_{z}\right)$ is the force, and $\Gamma =\left(\Gamma_{r},\Gamma_{z}\right)$ is the sensitivity.

The sensitivity $\Gamma =\left(\Gamma_{r},\Gamma_{z}\right)$ describes the magnitude of the derivatives of $F_{r}$ and $F_{z}$ with respect to $B_{r}$ and $B_{z}$, as given in Equations (1) and (2). For the sensor design optimisation, the objective was to minimise the change in the output of the sensor **F** for any given change in the input **B**, or in other words to minimise **Γ**. This ensured a robust sensor response, with **F** being less sensitive to noise in the measured **B**. Sensitivity was affected by the choices made in the design of the sensor such as the geometry, material properties and component selection. In the concept developed here, the magnet and Hall effect module were chosen leaving the geometry and material properties as design variables. This decision was made because it provides the simplest means of varying parameters in the design while demonstrating the complexity of delivering an optimised sensor.

(1) $$\Gamma_{r}=\sqrt{{\left(\frac{\partial F_{r}}{\partial B_{r}}\right)}^{2}+{\left(\frac{\partial F_{r}}{\partial B_{z}}\right)}^{2}}$$

(2) $$\Gamma_{z}=\sqrt{{\left(\frac{\partial F_{z}}{\partial B_{r}}\right)}^{2}+{\left(\frac{\partial F_{z}}{\partial B_{z}}\right)}^{2}}$$

#### 2.1.1. Sensor Mechanics

When the magnet was displaced by **u** the distance between the Hall effect module and magnet **Δ** was changed. The relationship between **u** and **Δ** was described by Equations (3) and (4),

(3) $$u_{z}=\Delta_{z}+H_{m}-H-H_{g}$$

(4) $$u_{r}=\Delta_{r}$$

where $u_{r}$ and $u_{z}$ are the displacement components, $\Delta_{z}$ and $\Delta_{r}$ are the r and z distances from the magnet to the Hall effect module, H is the sensor base height, $H_{m}$ is the height of the magnet, and $H_{g}$ is a geometrical height for the sensor. At a given $\Delta_{z}$ the reading of the Hall effect module became saturated, this limit $\Delta_{z,\mathrm{sat}}$. defined the depth to which the indentation reached. The magnet was displaced linearly with a non-dimensional variable t where 0≤t≤1, leading to Equations (5) and (6).

(5) $$u_{z}=t\left(\Delta_{z,\mathrm{sat}}+H_{m}-H-H_{g}\right)$$

(6) $$u_{r}=-t\left(\Delta_{z,\mathrm{sat}}+H_{m}-H-H_{g}\right)\tan\theta$$

The shear angle θ was limited to a value which was determined by an assumption derived from the frictional characteristics of the sensor and indenting surfaces. The coefficient of friction $\mu_{f}$ between the sensor and indenting surfaces was defined by Equation (7),

(7) $$\frac{F_{r,\mathrm{slip}}}{F_{z,\mathrm{slip}}}=\mu_{f}$$

where $F_{r,\mathrm{slip}}$ and $F_{z,\mathrm{slip}}$ are the shear and normal force components at slip. Beyond this limit the sensor material slips along the indenting surfaces, thus providing the limiting case under shear. Setting the ratio of displacement components equal to that of the force components at slip allowed an approximation for the maximum shear angle $\theta_{\max}$ to be determined, as described by Equation (8).

(8) $$\theta_{\max}=\tan^{-1}\mu_{f}$$

Structural mechanics simulations of the sensor under normal and shear loading, as described in Section 2.1.3, were conducted to ensure that this limit did not result in the elastomer material slipping along the indenting surface. The variables t and θ fully parameterised the range of displacements **u** required for a given sensor design. The space defined by the ranges of t and θ is continuous and bounded by t∈[0,1] and $\theta \in \left[0,\theta_{\max}\right]$.

#### 2.1.2. Magnetic Field

A model was developed to obtain the magnetic field in a fixed position and from which **B** was obtained as a function of the magnet displacement. This approach was applicable because the structural mechanics of the sensor under load does not have an effect on the magnetic field distribution. A 2D axially-symmetric cylindrical coordinate system was employed for developing the model because **B** is independent of the orientation of the shear direction r. The solution for **B** was described by Equation (9), and Equation (10) described the relationship between the magnetized field **H** and magnetic scalar potential V.

(9) ∇·B=0

(10) H=−∇V

**Figure 3:**
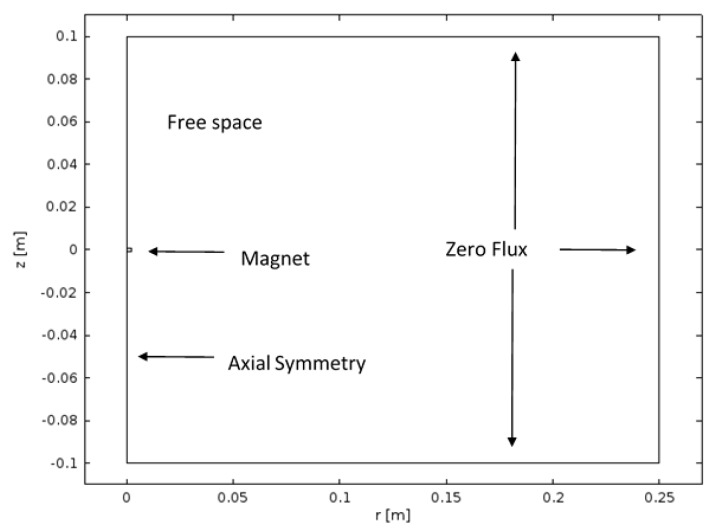
Diagram of the magnetic field model.

In the space surrounding the magnet, B was described by Equation (11) and within the magnet it was described by Equation (12),

(11) $$B=\mu_{0}\mu_{r}H$$

(12) $$B=\mu_{0}\left(H+M\right)$$

where $\mu_{0}$ is the permeability of free space, $\mu_{r}$ is the relative permeability, and M is the magnetisation as governed by the magnet selection. A zero flux condition, n·B=0 was applied at a boundary far from the sensor (+100 R_m_ in r and ± 50 H_m_ in z), where **n** is the surface normal vector and an axial-symmetry condition was applied through the z-axis of the magnet centre (r = 0), as shown in Figure 3. The magnetic field was not affected by the material properties of the elastomer such that the relative permeability of the elastomer and space surrounding the sensor is $\mu_{r}=1$, i.e., no shielding effect [27]. Using these operating and boundary conditions and solving Equations (9) and (10) together with Equations (11) and (12) for V produced the magnetic field B. This was subsequently assessed for all displacements and sensor designs by varying the distance between the magnet and assessment location.

#### 2.1.3. Structural Mechanics

During indentation the sensor material underwent large strains leading to a requirement for nonlinear structural mechanics to be used to describe the problem. The elastomer used was a rubber-like silicone (Ecoflex 00-30) for which the stress-strain relationship was known to be hyperelastic [28]. For the sensor design an incompressible Neo-Hookean model was used to define the material properties. Wang et al. [9] demonstrated that this model was sufficiently accurate to describe the mechanical behaviour of MagOne under load. The incompressible Neo-Hookean model derives the stress-strain relationship for the material from a strain energy density function W, as given in Equation (13),

(13) $$W=\frac{G}{2}\left(\lambda_{1}^{2}+\lambda_{2}^{2}+\lambda_{3}^{2}-3\right)$$

where G is the shear modulus of the elastomer, and $\lambda_{1}$, $\lambda_{2}$, $\lambda_{3}$ are the principle stretches. Stresses in the material can be obtained by calculating the derivatives of W with respect to strains, which themselves relate to the principle stretches. Variation of G can be achieved by changing the silicone used in the fabrication process, thus varying the elastomer stiffness. A steady-state assumption is made in the analysis such that no dynamic behaviour (viscoelasticity or inertia) was modelled. This was confirmed by experimental validation of the sensor showing 3.4% hysteresis [9].

**Figure 4:**
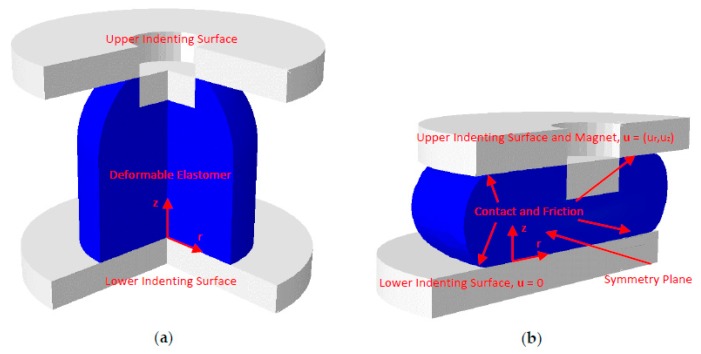
Diagram of the structural mechanics simulation domain. (**a**) Unloaded. (**b**) Loaded in both normal and shear directions.

In order to model indentation of the sensor to include both normal and shear loading a full three-dimensional description of the sensor was required, this was because as the sensor deformed in shear the shape of the sensor was not axially-symmetric, and thus, the dimension cannot be reduced as in the case for the magnetic field calculation. The magnet was modelled as a rigid body which was rigidly connected to the elastomer material surrounding it, and was displaced by $u_{r},u_{z}$ in the *r*-axis and *z*-axis, respectively, to produce normal and shear loads. The base of the elastomer was fixed such that the displacement was constrained to zero u=0, and the sides of the sensor were allowed to freely deform. A symmetry condition was specified in the plane to which shear was applied. Contact mechanics was considered to model the interaction between the freely deforming surfaces of the elastomer and the indenting surfaces, which were themselves considered rigid bodies. Friction was generated during indentation and this effect was modelled by specifying a coefficient of friction $\mu_{f}$ for the contact. Figure 4a,b show the structural model of the sensor for when it was unloaded and loaded, respectively. Equation (13) was solved according to these operating and boundary conditions to give the stress distribution under load over the required ranges of displacement and sensor designs. The value of $F=\left(F_{r},F_{z}\right)$ was then given by determining the reaction force at the lower indenting surface.

### 2.2. Design Specification

For the design optimisation of MagOne two variables were investigated: (i) the sensor base height H, and (ii) the elastomer shear modulus G. Together these variables describe the sensor design in terms of geometry and material properties. It is feasible to fabricate sensors over reasonable ranges of the height H and the shear modulus G making them appropriate design variables for an optimisation study. Any number of parameters could be selected as design variables for the sensor, including the choice of components not investigated here, but H and G have been chosen to demonstrate how this type of sensor can be optimised by design. The ranges used for each of the design variables were chosen as H∈[3,7] mm and G∈[1,5] kPa as to span reasonable values of the identified variables, the sensor designed by Wang et al. [9] used H = 3 mm and G = 2.94 kPa. The remaining parameters required for the sensor design and mechanics are given in Table 1.

#### 2.2.1. Parameterisation

The magnetic field B and force **F** were determined over the range of the design variables and displacements. The responses were subsequently characterised by Equations (14) and (15) as functions of the parameters G, H, t, and θ. The sensitivity **Γ** was calculated by taking partial derivatives of **F** with respect to B, leading to Equation (16).

(14) B=f(t,θ,H)

(15) F=f(B,t,θ,G,H)

(16) Γ=f(B,t,θ,G,H)

GP was used to derive phenomenological expressions representing each of Equations (14) and (15), and from which Equation (16) was subsequently determined. The form of these expressions used any mathematical operator with any combination of the input variables. The expressions were derived based on an evolutionary bio-inspired algorithm similar to that used in GAs for optimisation problems [29], which accurately describe complex non-linear trends in the response that were not simple (or even possible) to derive from first principles [30]. Due to the non-linearity associated with the magnetic field and force responses of MagOne under load, GP provided a useful means for obtaining the algebraic relationships required [20].

#### 2.2.2. Loading Stability

**Figure 5:**
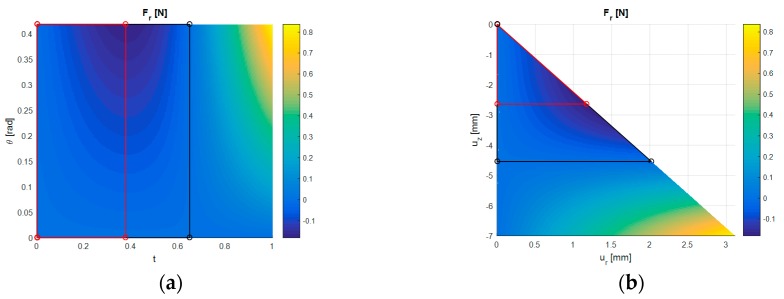
Response of $F_{r}$ in N for H = 7 mm and G = 5 kPa. Region of stable shear loading bounded in red, point placed at the location the minimum $F_{r}$. Region of negative $F_{r}$ bounded in black, point placed at the location of $F_{r}=0$ for $\theta =\theta_{\max}$. (**a**) $F_{r}$ shown as a function of t and θ. (**b**) $F_{r}$ shown as a function of $u_{r}$ and $u_{z}$.

As the sensor is displaced in both the normal and shear directions it is known that the normal force $F_{z}$ will monotonically increase; however, the shear force $F_{r}$ does not exhibit the same type of response and as such the stable region for sensing where an increase in displacement correlates to an increase in $F_{r}$ is therefore reduced. The effect of t and θ on $F_{r}$ is shown in Figure 5a and the effect of $u_{r}$ and $u_{z}$ on $F_{r}$ is shown in Figure 5b for when H = 7 mm and G = 5 kPa. The region indicated in black identifies where $F_{r}$ is always monotonically decreasing with $u_{r}$ for any given $u_{z}$, which itself corresponds to when $F_{r}$ is negative in value. Further to this, the region identified in red shows the range of displacements for when $F_{r}$ is monotonically decreasing from zero to the minimum value $F_{r,\min}$. This region can be considered stable under shear loading because it ensures that both the shear force and normal force are monotonically changing with displacement and reach peak values at the same instance.

With shear loading considered in the design this stability condition was imposed and the bounds of the feasible displacements reduced to $t^{\prime }\in \left[0,t_{m}\right]$ and $\theta^{\prime }\in \left[0,\theta_{m}\right]$, where $t_{m}$ and $\theta_{m}$ are the values of t and θ corresponding to $F_{r,\min}$ for any given value of H and G (see Equation (17)). The optimisation is performed within these bounds (see Section 2.2.3) but this does not limit the operation of the sensor, only the range of stable displacements which the design is optimised for. Corresponding to these reduced bounds, the maximum normal force $F_{z,\max}$ and maximum shear/normal sensitivities $\Gamma_{r,\max},\Gamma_{z,\max}$ can be determined for any given value of H and G by Equation (18).

(17) $$\mathrm{minimise}:\;F_{r}\;\mathrm{subject}\;\mathrm{to}:\;0\leq t\leq 1,\;0\leq \theta \leq \theta_{\max}\;\mathrm{to}\;\mathrm{yield}:\;F_{r,\min}\left(t_{m},\theta_{m}\right)=f\left(H,G\right)$$

(18) $$\mathrm{maximise}:\;F_{z},\Gamma_{r},\Gamma_{z}\;\mathrm{subject}\;\mathrm{to}:\;0\leq t\leq t_{m},\;0\leq \theta \leq \theta_{m}\;\mathrm{to}\;\mathrm{yield}:\;F_{z,\max},\Gamma_{r,\max},\Gamma_{z,\max}=f\left(H,G\right)$$

#### 2.2.3. Design Optimisation

The optimisation objective for the sensor design was to minimise the sensitivity **Γ** over the ranges of the design variables H and G. In order to achieve this the worst sensitivity (maximum value of **Γ**) over all stable displacements was used. The worst sensitivity corresponds to the maximum value of **Γ** because this implies the greatest rate of change of **F** with the measured **B**. By minimising the worst sensitivity achieved during displacement ensures that the optimised design has the best possible sensitivity for all displacements considered. By using the stable range of displacements the optimised sensor was also ensured to produce a monotonically changing **F** with measured **B**.

For each case a measureable maximum/minimum force $F_{c}=\left(F_{r,c},F_{z,c}\right)$ was specified which subsequently constrained the optimisation such that this force could at least be measured. These force constraints ensured that the minimum possible shear force the sensor measured $F_{r,\min}$ was less than or equal to the constrained value $F_{r,c}$, and the maximum possible normal force the sensor measured $F_{z,\max}$ was greater than or equal to the constrained value $F_{r,z}$. Given that both force components were constrained by design, the objective for the optimisation corresponded to minimising the worst sensitivity in both directions (that is minimising $\Gamma_{r,\max}$ and $\Gamma_{z,\max}$) as described by the multi-objective problem, Equation (19). This produced optimal values of the design variables $H^{*}$ and $G^{*}$ with corresponding obejctives $\Gamma_{r,\max}^{*}$, $\Gamma_{z,\max}^{*}$ and constraints $F_{r,\min}^{*}$, $F_{z,\max}^{*}$.

(19) $$\mathrm{minimise}:\;\Gamma_{r,\max}\;\mathrm{and}\;\Gamma_{z,\max}\;\mathrm{subject}\;\mathrm{to}:\;3\leq H\leq 7\;\left[\mathrm{mm}\right],\;1\leq G\leq 5\left[\mathrm{kPa}\right]\;F_{r,\min}-F_{r,c}\leq 0,{\;F}_{c,z}-F_{z,\max}\leq 0\;\mathrm{to}\;\mathrm{yield}:\;\Gamma_{r,\max}^{*},\;\Gamma_{z,\max}^{*},F_{r,\min}^{*},F_{z,\max}^{*}=f\left(H^{*},G^{*}\right)$$

### 2.3. Numerical Simulations

#### 2.3.1. Magnetic Field Simulation

In order to calculate the magnetic field components $B_{r},B_{z}$, Equations (9)–(12) were solved and from which **B** was determined in a fixed cylindrical coordinate system according to the boundary conditions outlined in Section 2.1.2. This was undertaken using the FE method as implemented in the software COMSOL Multiphysics [31]. The magnetisation **M** was chosen based on the characteristics specified by the manufacturer and the orientation of the magnetic poles. For the sensor concept developed by Wang et al. [9] $M_{z}=1.2\times 10^{6}$ A/m and the remaining components were set to zero. The permeability of free space was given by $\mu_{0}=1.257\times 10^{-6}$ (m.kg)/(s.A)^2^.

In the simulation 18,555 second-order triangular elements were used to discretise the domain, with the smallest elements placed on the magnet and grown in size toward the external boundary. This number of elements was shown to be significant in producing grid independent results, with an increase in accuracy in the measurement of **B** of less than 1.34% produced when a greater number of elements was used. The calculation took approximately 2 min to run on a 2.8 GHz 4-core CPU with 16 GB of RAM. Once the solution was achieved a post-processing stage allowed **B** to be given as a function of t, θ, H from the fixed position solution.

#### 2.3.2. Structural Mechanics Simulations

The shear and normal forces $F_{r},F_{z}$ were calculated using a model developed with Abaqus CAE [32] which employs the FE method. The incompressible Neo-Hookean hyperelastic model was specified for the material properties, and the boundary conditions implemented were according to those outlined in Section 2.1.3. The magnet and indenting surfaces were represented by rigid bodies such that they did not deform under load, and the elastomer was rigidly connected to the magnet surfaces such that they had the same displacement. The penalty contact algorithm was used to describe the normal contact pressures due to the interaction of the elastomer and rigid bodies. Tangential contact tractions were modelled by assigning a coefficient of friction $\mu_{f}=0.45$, which is a known value for rubber-like silicone on a hard surface and represents frictional behaviour similar to that of human skin [33]. The standard settings for the explicit (time-dependent) solver were used. The time period for indentation (1 s) was assumed to be large enough for inertia to have no dynamic effect and as such the results produced were quasi-static.

The number of elements used in the structural mechanics simulations varied because the geometry was parameterised by the variable H, therefore a minimum and maximum element size were chosen to use across all geometries. Minimum and maximum element length scales of 7.5 µm and 100 μm were used, respectively, and the resulting discretised domains varied in number from 127,877 to 285,150 second-order tetrahedral elements over the range of H. These values were found to produce grid-independent results with an increase in accuracy in the measurement of **F** of less than 1.88% found when using smaller element sizes. The meshing procedure ensured that the smallest elements were placed in regions where high stress and strains are produced under load, as such the corner and edge regions on the domain were more densely populated than regions far from boundaries. For each value of θ, G, H a simulation was run and $F_{r},F_{z}$ extracted as a function of t from the lower (stationary) indenting surface. The time to compute varied based on the values of θ, G, H. The longest simulation occurred at the maximum for each parameter and took approximately 3 h 40 min using the same computer hardware as described in Section 2.3.1.

#### 2.3.3. Genetic Programming

The open source toolbox GPTIPS [34] was used to derive phenomenological expressions representing Equations (14) and (15) by GP. The toolbox is written using the Matlab [35] programming language. The data required by GPTIPS to calculate these expressions must span the ranges of t, θ, G, and H; additionally, the corresponding values of **B** and **F** must be calculated from the simulations as described in Section 2.3.1 and Section 2.3.2. For this purpose a full factorial Design of Experiments (DOE) was used to select the values of t, θ, G, and H as to ensure that the entire design space was populated in an evenly-distributed manner. The number of experiments in each dimension were chosen as: 21 in t; 5 in θ; 5 in H; and 5 in G. The total number of DOE points in the **B** response was 525 which were obtained from 1 simulation, and the total number of DOE points in the **F** response was 2625, obtained from 125 simulations (taking approximately 2 weeks to compute using the same computer hardware as outlined in Section 2.3.1).

For each of the expressions generated by GPTIPS the types of mathematical operators which can be used is controlled by the user. The combination of these as functions of the input variables is chosen to best represent the output data via the use of an evolutionary algorithm [20]. In the case of deriving equations to represent Equations (14) and (15), the operators were limited to those which can be continuously differentiated so that the derivatives also take analytical forms and can be used in the optimisation procedure, as outlined in Section 2.3.4. The GPTIPS algorithm also allowed control of parameters relating to the number of genes (or component part of each expression), the complexity of each gene, population size, and solution tolerances. An increase in the number of genes, their complexity, and the population size or decrease in the solver tolerances will increase the likelihood that a more accurate expression is generated; however, this becomes a trade-off with the length of time which the solution takes. In each instance that GPTIPS fits an expression to a data set it is likely that a different solution will be generated. This is because the total number of different combinations of the input and mathematical expressions is very large and by random number generation it is unlikely that the same combinations will be produced. In the case of Equations (14) and (15) the solver tolerances were reduced to 10^−9^ and the remaining parameters set to their default values. The solver was subsequently run for 4 h to derive each of the expressions for $B_{r},B_{z},F_{r},$ and $F_{z}$. These tolerances and time to compute has been used in previous studies to obtain sufficiently accurate relationships [25]. After calculating these expressions they were differentiated using Matlab to provide the sensitivity $\Gamma_{r},\Gamma_{z}$ (Equation (16)) and higher derivatives needed for the optimisation studies.

#### 2.3.4. Optimisation Procedure

The optimisation studies were conducted using the Matlab optimisation toolbox [35]. For each study the objective formed a minimax type problem with nonlinear constraints [36]. The minimax condition was satisfied by solving for $\Gamma_{r,\max}$ and $\Gamma_{z,\max}$ as functions of H and G and then subsequently minimising this value. Similarly, $F_{r,\min}$ and $F_{z,\max}$ were determined as functions of H and G for use in the constraints. Underlying each of these optimisation studies was a sub-optimisation sequence in which $\Gamma_{r,\max},{\;\Gamma }_{z,\max},{\;F}_{r,\min}$ and $F_{z,\max}$ were determined as functions of t and θ. Therefore, each optimisation study was separated into two parts: (i) unconstrained optimisation of sensitivity and force to find the maximum/minimum values for all displacements (Equations (17) and (18)), and (ii) force-constrained optimisation of sensitivity to find the minimum values for all design variables (Equation (19)).

In (i) values of H and G were specified and for which $\Gamma_{r,\max},\;\Gamma_{z,\max},\;F_{r,\min}$ and $F_{z,\max}$ are to be determined as functions of t and θ. This was achieved using a GA in combination with a heuristic-based optimisation solver. The GA was used to find values of t and θ which give the initial guess of the heuristic solver; this process ensured that the minimum identified by the GA was refined to the exact global minimum [21]. Where a maximum was to be found the identity $\underset{x}{\max}\left(f\left(x\right)\right)=-\underset{x}{\min}\left(-f\left(x\right)\right)$ was used. The Matlab function *ga* was implemented to find the initial guess for the heuristic solver which was itself the Matlab function *fmincon*, for both all tolerances were set to 10^−12^. For *ga* the population size was set to 200, and within *fmincon* the trust-region-reflective-algorithm was chosen to which the gradients and Hessian of **Γ** and **F** with respect to t and θ were supplied. Due to stability conditions, $F_{r,\min}$ and the corresponding $t_{m}$ and $\theta_{m}$ were solved for first because $t^{\prime }$ and $\theta^{\prime }$ were required for the calculation of the $\Gamma_{r,\max},\Gamma_{z,\max}$ and $F_{z,\max}$.

A GA was used in (ii) to find the minimum values of $\Gamma_{r,\max}$ and $\Gamma_{z,\max}$ returned from (i) as functions of H and G. This was subject to force constraints for which $F_{r,\min}$ and $F_{z,\max}$ were also given from (i) as functions of H and G. The Matlab function *gamultiobj* was used in combination with nonlinear constraints to solve the multi-objective optimisation problem. An equal weighting was applied to the objective function components in order to generate a single cost function which the optimisation algorithm minimises. This subsequently produced a Pareto set of designs which minimise both components without bias. Each part of the Pareto set is equally optimal and a decision making process was required to establish the optimal design solution $\Gamma_{r,\max}^{*},{\;\Gamma }_{z,\max}^{*},F_{r,\min}^{*},F_{z,\max}^{*}=f\left(H^{*},G^{*}\right)$. In each case all tolerances in the solver were specified as 10^−12^ and the population size was set to 200. For the purpose of this study the force constraints were specified as $F_{r,c}=-0.25\;N$ and $F_{z,c}=5\;N$, respectively. The optimisation procedure and corresponding data required for visualisation took approximately 23 h 35 min to compute.

**Figure A1:**
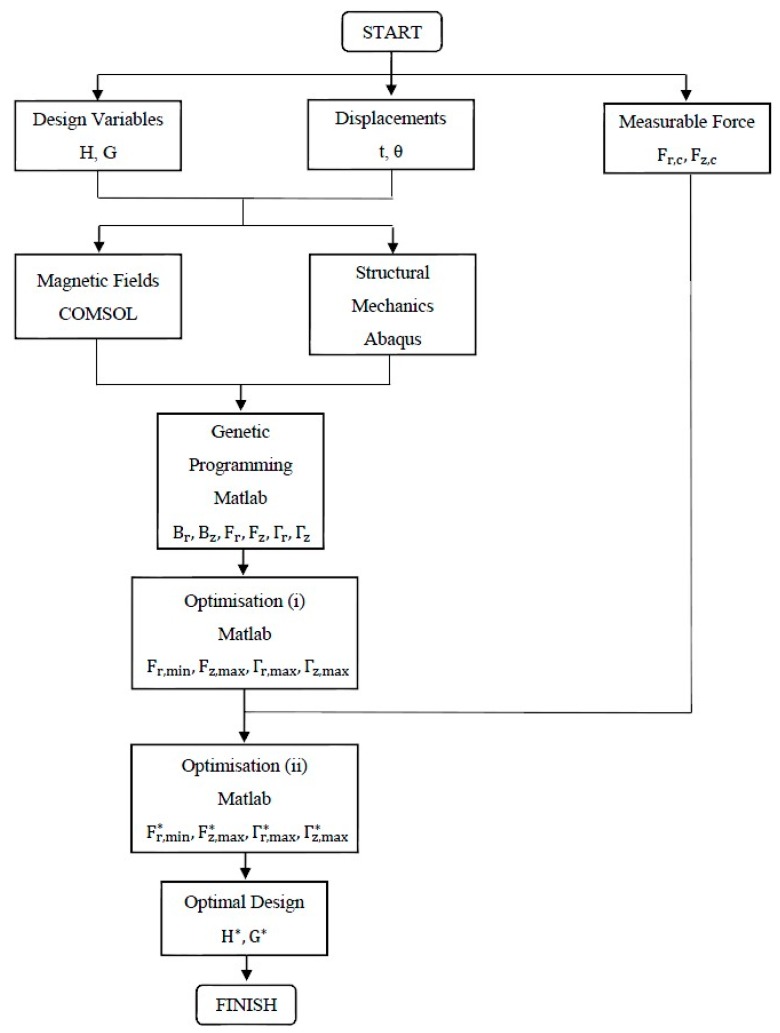
A flow chart of the optimisation procedure developed.

A flow chart outlining the optimisation procedure developed in this work is given in Appendix A (Figure A1).

### 2.4. Design Validation

In order to validate the optimised design a set of four new sensors were fabricated according to the optimised sensor height $H^{*}$ and material stiffness $G^{*}$, as given by the result of the method described in Section 2.3.4. To achieve this a mould was printed using stereolithography at a 25 μm resolution [37]. The mould was then cast with a silicone elastomer [38] and left to cure at room temperature. Once cured, magnets were embedded into the nodes of the sensor bodies with a silicone adhesive [39]. After ensuring that the magnets were fully encapsulated into the sensor bodies, the sensors were mounted onto custom designed 3D printed circuit boards for experimental testing [9].

The new sensors (Figure 1b) were tested experimentally through a custom test platform consisting of a force/torque (F/T) sensor [40], mounting brackets, the magnetic sensor and two motorised linear stages [41] positioned to provide compression in the *z*-axis and shear force about the *x*-axis. The linear stages had a minimum step of 0.01 mm, travel range of 75 mm, and repeatability of 2.5 µm, while the F/T sensor has a measuring range of ±35 N in the z-axis, ±25 N in *x*/*y*–axis, and a resolution of 6.25 mN in all axes. A custom program was developed using LabView [42] to calibrate and control the movement of the motorised linear stages; this was used to digitally acquire the measurements from the F/T sensor and the magnetic sensor. The step size for the linear stage in all axes was set to the minimum 0.01 mm and the total time taken for each of the indentation tests was ~3 h. This allowed for a high resolution data capture and also assisted in minimising transient and slip effects of the sensor under load. With the aim of ensuring that the observed experimental behaviour is as close as possible to the steady-state assumptions underpinning the simulated response.

Two repeats were made for each indentation test conducted on each of the four newly fabricated sensors, this number was used to ensure that the results obtained were a true representation of the repeatability of the sensor. Results generated from each repeat test were then processed using a high-band/low-band filter (available from the online Matlab file exchange [43]) in order to reduce noise in the experimental data. For this purpose, the raw data was sent through a Fourier transform and subsequently all frequencies above and below given limits were cut-off; the data was then converted back to the original space using the inverse Fourier transform operation. Default settings for the cut-off bandwidths were specified in the software. Using the filtered data, the mean and standard deviation of the force and magnetic field responses were calculated across all repeats as a function of the indentation displacement using Equations (20) and (21), respectively. Where N=8 (4 sensors times 2 repeats) is the total number of indentation tests, φ are the parameters describing indentation (displacement for a given shear angle), $\psi_{i}$ are the response variables (force and magnetic field) for the I’th repeat test, $\psi_{\mu }$ is the mean of the response variables over the number of repeats, and $\psi_{\sigma }$ is the standard deviation of the response variables over the number of repeats. In order to assess the variability in the experimental responses the maximum coefficient of variation $c_{v,\psi }$ over the full range of displacements was calculated using Equation (22). This gives a measure for the variation about the mean value obtained, with $c_{v,\psi }$ approaching zero implying that the mean is close to all values obtained over the number of repeats and displacements considered.

(20) $$\psi_{\mu }\left(\varphi \right)=\frac{1}{N}\overset{i=N}{\underset{i=1}{\sum }}\psi_{i}\left(\varphi \right)$$

(21) $$\psi_{\sigma }\left(\varphi \right)=\sqrt{\frac{1}{N}\overset{i=N}{\underset{i=1}{\sum }}{\left(\psi_{i}\left(\varphi \right)-\psi_{\mu }\left(\varphi \right)\right)}^{2}}$$

(22) $$c_{v,\psi }=\underset{\varphi }{\max}\left|\frac{\psi_{\sigma }\left(\varphi \right)}{\psi_{\mu }\left(\varphi \right)}\right|\times 100\%$$

The mean values obtained experimentally were subsequently compared to those generated from GP under the same conditions. This was undertaken by calculating the maximum absolute percentage error between the mean experimental and computational results over the range of displacements, as described by Equation (23). Where $\epsilon_{\psi }$ are the maximum absolute percentage errors and ψ are the response variables obtained computationally from GP. An analysis was then made investigating the sensitivities of the optimised and original designs, in which the percentage difference between the two sets of data obtained from GP for these conditions were calculated and compared.

(23) $$\epsilon_{\psi }=\underset{\varphi }{\max}\left|\frac{\psi_{\mu }\left(\varphi \right)-\psi \left(\varphi \right)}{\psi \left(\varphi \right)}\right|\times 100\%$$

## 3. Results

### 3.1. Magnetic Field

**Figure 6:**
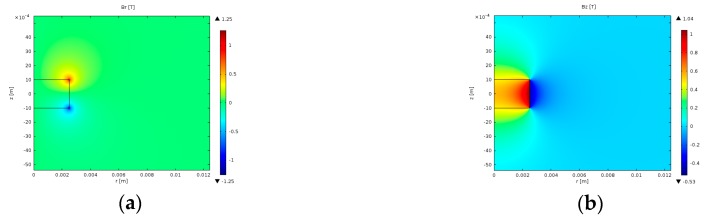
Magnetic field distribution in the near magnet region. (**a**) Coloured by $B_{r}$. (**b**) Coloured by $B_{z}$.

Figure 6a,b illustrate $B_{r}$ and $B_{z}$ distributions, respectively, calculated from the 2D axially-symmetric FE simulation of the magnetic field as described in Section 2.3.1. Each figure shows the magnetic field components in the region close to the magnet by means of filled, coloured contours in units of Tesla. It is demonstrated that the strength of $B_{r}$ and $B_{z}$ diminishes with increasing distance from the magnet in both the r and z directions. Figure 6b shows that the distribution of $B_{z}$ is symmetric about the centre of the magnet at z = 0 mm, and Figure 6a shows that $B_{r}$ is anti-symmetric about the centre of the magnet where z = 0 mm. The responses generated by GP as a result of the magnetic field simulation were of a high level of accuracy, with the errors in $B_{r}$ and $B_{z}$ shown to be two orders of magnitude smaller than the values obtained from FE.

### 3.2. Strcutural Mechanics

**Figure 7:**
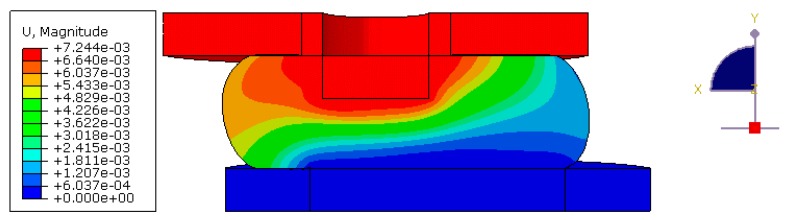
Response of displacement magnitude shown in m for the sensor under load at t = 1, θ = 0.423 rad, H = 7 mm and G = 5 kPa.

An example of the structural mechanics results of the MagOne sensor under normal and shear loading is presented in Figure 7, which illustrates the distribution of displacement magnitude in the elastomer at t = 1, θ = 0.423 rad, H = 7 mm and G = 5 kPa. Due to normal loading the elastomer material is compressed in the z-direction between the rigid surfaces which due to the boundary conditions imposed results in ± displacement of material in the r-direction. Material is displaced further in the positive r-direction than the negative because this is the shear loading orientation. It is also shown that under load the elastomer contacts the upper and lower indenting surfaces and since material cannot deform past these locations the shape of the sensor is significantly changed. The responses generated by GP as a result of the structural mechanics simulations were accurate, with the errors in $F_{r}$ and $F_{z}$ an order of magnitude smaller than the values obtained from FE over the full range of t and θ. The shear force $F_{r}$ obtained from the structural mechanics simulations as a function of the displacements $u_{r}$ and $u_{z}$ is presented in Figure 5a and as a function of t and θ in Figure 5b, corresponding to this the normal force $F_{z}$ calculated monotonically increased with t and remained constant for all θ.

### 3.3. Sensitivity

**Figure 8:**
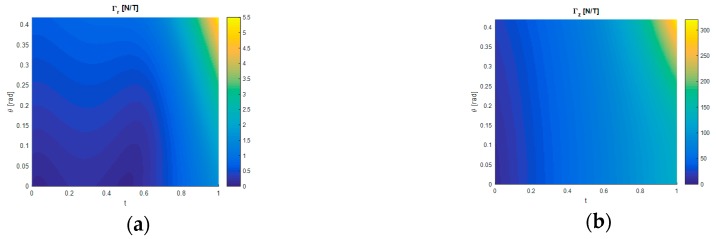
Sensitivity responses generated from GP showing the effect of t and θ for H = 7 mm and G = 5 kPa. (**a**) Coloured by $\Gamma_{r}$. (**b**) Coloured by $\Gamma_{z}$.

An example of the sensitivity components $\Gamma_{r}$ and $\Gamma_{z}$ produced under load are given in Figure 8a,b, respectively, for H = 7 mm and G = 5 kPa. Each relationship is derived from the force and magnetic field equations generated by GP and are shown as functions of the displacement described by t and θ. Because the results presented in Section 3.1 and Section 3.2 are shown to be accurate it follows that the sensitivity is also accurately described. Figure 8a,b show that the sensitivity has a non-linear response as a function of t and θ which can only be described by the solutions obtained computationally. Figure 8a illustrates that $\Gamma_{r}$ tends to increase with increasing t but the response is not always monotonic. $\Gamma_{r}$ increases with increasing θ and the response is monotonic. Figure 8b shows that $\Gamma_{z}$ increases monotonically with both t and θ and is an order of magnitude larger than $\Gamma_{r}$.

### 3.4. Design Optimisation

**Figure 9:**
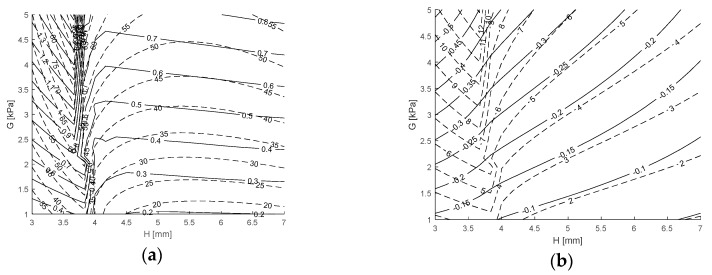
Response of the objectives (**a**) and constraints (**b**) as functions of H and G under stable shear loading conditions. (**a**) Showing contours of $\Gamma_{r,\max}$ in N/T (solid) and $\Gamma_{z,\max}\;$ in N/T (dashed). (**b**) Showing contours of $F_{r,\min}$ in N (solid) and $F_{z,\max}$ in N (dashed).

Figure 9a,b show under stable shear loading conditions, the responses of the objectives $\Gamma_{r,\max}$ and $\Gamma_{z,\max}$ and responses of the constraints $F_{r,\min}$ and $F_{z,\max}$ as functions of the design variables H and G, respectively. Both Figure 9a,b were generated by assessing the sub-optimisation procedure (i) as described in Section 2.3.4 over a 10 × 10 grid of H and G values, and subsequently linearly interpolated to produce the result between the known locations. Figure 9a shows that there is a nonlinear response of $\Gamma_{r,\max}$ and $\Gamma_{z,\max}$ as functions of H and G. Toward the bounds of H both functions are shown to decrease with increasing H, whereas, in the regions near H = 4 mm there are sharp turning points where both functions suddenly increase with increasing H. The effect of increasing G increases both functions. Figure 9b shows that $F_{z,\max}$ has a similar type of response as $\Gamma_{r,\max}$ and $\Gamma_{z,\max}$ over the ranges of H and G, whereas, $F_{r,\min}$ decreases monotonically with increasing H and increases monotonically with increasing G. The turning points identified correspond to the values of H and G for which $F_{r,\min}$ is no longer at the maximum t and θ, that is, when shear loading becomes unstable and there is a change in the bounds, t and θ from the measurements of $F_{z,\max},{\;\Gamma }_{r,\max}$ and $\Gamma_{z,\max}$ are refined.

**Figure 10:**
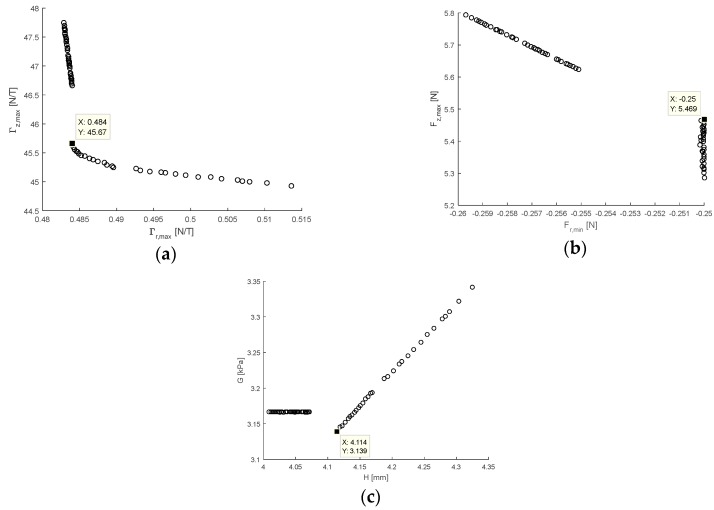
Pareto optimal set showing: (**a**) the competing objectives of $\Gamma_{r,\max}$ and $\Gamma_{z,\max}$; (**b**) the competing constraints of $F_{r,\min}$ and $F_{z,\max}$; and (**c**) the design variables H and G. Result obtained under stable loading conditions with $F_{c,r}$ = −0.25 N and $F_{c,z}$ = 5 N.

The multi-objective optimisation procedure (ii) as described in Section 2.3.4 was conducted with force constraints $F_{r,c}=$ −0.25 N and $F_{z,c}=5\;N$ used as an example. The Pareto optimal set of objectives generated as a result of the optimisation is presented in Figure 10a, and subsequently Figure 10b,c present the corresponding constraints and design variables. The Pareto set was shown to be disconnected in the design space due to the competing objectives and constraints with two regions identified: (i) where the $\Gamma_{z,\max}$ changed linearly with the $\Gamma_{r,\max}$; and (ii) where the $\Gamma_{r,\max}$ changed with an increasing rate with $F_{z,\max}$. In (i) $F_{r,\min}$ decreases linearly with $F_{z,\max}$ and both constraints are satisfied, also G is constant for all H. Whereas, in (ii) $F_{r,\min}$ is always equal to the constraint value for all $F_{z,\max}$ and the value of G produced increases linearly with H.

The optimal sensor design under shear loading was determined by assuming equal weighting of the solutions over the norms of the Pareto set and selecting the design which produced the lowest value. This corresponded to the lowest value of $\Gamma_{r,\max}$ and highest value of $\Gamma_{z,\max}$ in region (ii). The optimal design and corresponding objectives, constraints, and design variables are highlighted in Figure 10a–c, respectively. Under stable loading conditions and the specified force constraints, the optimal sensor design was selected as $H^{*}$ = 4.11 mm and $G^{*}$ = 3.14 kPa. This corresponded to $\Gamma_{r,\max}^{*}$ = 0.48 T/N and $\Gamma_{z,\max}^{*}$ = 45.7 T/N, with $F_{r,\min}^{*}$ = −0.25 N and $F_{z,\max}^{*}$ = 5.47 N.

This result compares well to the sensitivities of $\Gamma_{r,\max}$ = 0.81 T/N and $\Gamma_{z,\max}$ = 50.5 T/N for the design as originally proposed by Wang et al. [9] where H = 3 mm and G = 2.94 kPa. It is of note that their design did not consider the force constraints as imposed by the optimisation such that for their design $F_{r,\min}$ = −0.34 N and $F_{z,\max}$ = 7.03 N; this further demonstrates how the optimised design improves sensitivity at the expense of the constrained measureable force. Setting the constraint force to $F_{r,c}=$ −0.34 N and $F_{r,c}=7.03\;N$ and evaluating the optimisation study again yielded optimal values of $H^{*}$ = 4.17 mm and $G^{*}$ = 4.51 kPa, which gives a taller and stiffer sensor than that of Wang et al. [9] for the same measureable force. For this design $\Gamma_{r,\max}^{*}$ = 0.68 T/N and $\Gamma_{z,\max}^{*}$ = 56.7 T/N showing that for the optimal sensor shear sensitivity has comparatively been reduced at the expense of normal sensitivity. Corresponding to this $F_{r,\min}^{*}$ = −0.34 N and $F_{z,\max}^{*}$ = 7.15 N, indicating that only the shear loading constraint is active for the optimal design.

### 3.5. Design Validation

A set of optimised sensors were fabricated and tested using the method described in Section 2.4, the sensor height was chosen as H = 4.1 mm and the material had the stiffness of the original sensor G = 2.94 kPa which is a 6.8% difference of the optimised value. This height and stiffness were selected to simplify the fabrication process, with considerable further research required in order to adjust these parameters more precisely. Indentation tests were conducted for the maximum shear angle $\theta_{\max}$. After undertaking repeat indentation tests on the set of new sensors the mean and standard deviations for the magnetic field and force were determined as functions of the displacement. Using this data, the maximum coefficient of variation for each of the measured responses were determined as the following: $c_{v,B_{r}}=5.4\%$; $c_{v,B_{z}}=3.2\%$; $c_{v,F_{r}}=9.4\%$; $c_{v,F_{z}}=8.7\%$. This variability is less than 10% of the mean in all cases and indicates that the mean values obtained from the experimental testing procedure are a good approximation of the sensor response over the number of repeats and range of displacements considered.

**Figure 11:**
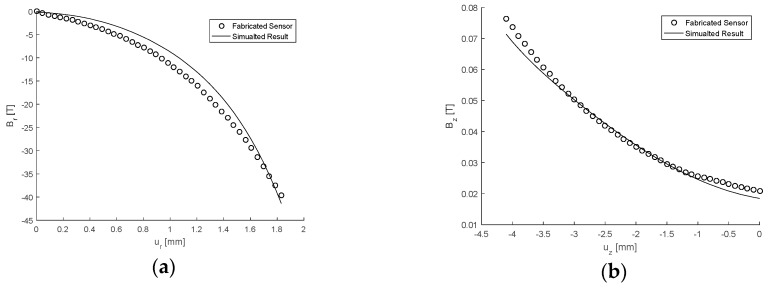
Response of the magnetic field components given as a function displacement with $\theta =\theta_{\max}$, H = 4.1 mm and G = 2.94 kPa. (**a**) Showing $B_{r}$ as a function of $u_{r}$ as simulated and from the experimental mean. (**b**) Showing $B_{z}$ as a function of $u_{z}\;$ as simulated and from the experimental mean.

**Figure 12:**
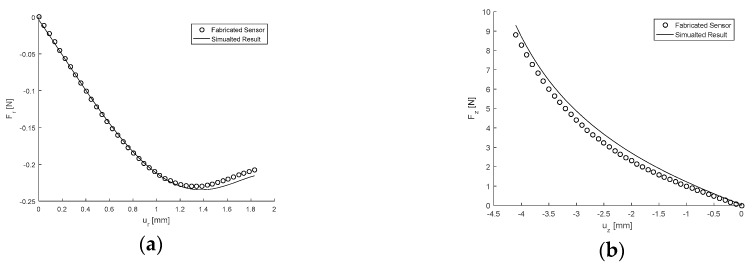
Response of the force components given as a function of displacement with $\theta =\theta_{\max}$, H = 4.1 mm and G = 2.94 kPa. (**a**) Showing $F_{r}$ as a function of $u_{r}$ as simulated and from the experimental mean. (**b**) Showing $F_{z}$ as a function of $u_{z}$ as simulated and from the experimental mean.

The magnetic field components $B_{r}$ and $B_{z}$ obtained from the experimental mean and as simulated under the same conditions are presented in Figure 11a,b as functions of the displacements $u_{r}$ and $u_{z}$, respectively. Corresponding to this the force, components $F_{r}$ and $F_{z}$ are given in Figure 12a,b. The maximum absolute percentage errors between the mean experimental results and those generated using GP were determined and it was found for the magnetic field components that $\epsilon_{B_{r}}=6.8\%$ and $\epsilon_{B_{z}}=3.4\%$, indicating the experimental results are within 7% of the measurements determined computationally. Similarly for the force components the errors were calculated as $\epsilon_{F_{r}}=4.7\%$ and $\epsilon_{F_{z}}=4.1\%$ which shows that the experimental results are within 5% of the corresponding computational data. Therefore, for the case considered, the computational models and use of GP are a sufficiently accurate means of describing the sensor mechanics. Subsequently this also provides evidence that the optimisation conducted using the simulated results are reliable. However, further experimentation in other regions of the design space would need to be conducted to ensure this is true for any design specification which can be conceptualised.

For the design fabricated, the maximum sensitivities were calculated using the computational results to be $\Gamma_{r,\max}$ = 0.47 T/N and $\Gamma_{z,\max}$ = 43.8 T/N, with the corresponding measureable forces given as $F_{r,\min}$ = −0.23 N and $F_{z,\max}$ = 5.23 N. This represents a 41.2% and 13.3% reduction in the maximum sensitivities $\Gamma_{r,\max}$ and $\Gamma_{z,\max}$, respectively, when compared to that of the original design [9] when calculated from the computational model. This is achieved with a 32.4% increase and a 25.9% reduction in the measureable forces $F_{r,\min}$ and $F_{r,\max}$, corresponding to a 36.7% increase in the sensor height H. Therefore, based on the simulated results alone it is shown that the fabricated design is a significant improvement in terms of sensitivity on the original design, and that this is achieved at the cost of the stable force range in which the sensor can measure.

## 4. Discussion

This paper investigates the design optimisation of a magnet field based tactile soft sensor as originally designed by Wang et al. [9]. The sensor design is optimised for sensitivity in terms of the geometry and material properties subject to stable loading conditions and measurable force constraints. The design variables selected consisted of the sensor base height H which described the geometry, and the elastomer shear modulus G which described the material properties. Sensitivity was defined as the rate of change of the sensor output (force) with input (magnetic field), and for which the worst case (highest value) was to be minimised in the optimisation. Stable loading conditions ensured a monotonic change in force with displacement, and constraints imposed ensured that the sensor was capable of measuring a specified force.

FE simulations were employed to obtain solutions to the magnetic field and structural behaviour of the parameterised sensor design under load. GP was used to generate phenomenological expressions underlying the responses of the sensor and were shown to be accurate representations of the FE simulations to within an order of magnitude. The optimisation procedures employed consisted of two steps: (i) unconstrained single-objective optimisation (solved with a GA and heuristic solver); and (ii) multi-objective optimisation with nonlinear constraints (solved with a GA). This produced a Pareto optimal set of sensor designs and from which a decision making process was employed to yield the optimised design. It was shown the design is optimised to minimise sensitivity, but this was achieved at the expense of the measureable force, and further that for the same conditions as originally proposed by Wang et al. [9] a taller and stiffer design would be optimal. Validation of the optimal design delivered was conducted by fabricating and testing a new sensor, the results showed that under the prescribed conditions the optimisation procedure was accurate in predicting the performance of the sensor, and that this was an improvement compared to the original design. It is also highlighted that in order to validate results across the full range of variables investigated, further testing would be required.

The method developed in this paper provides an accurate means of describing the sensor mechanics in operation and indicates how to optimise parameters in the design to improve the sensitivity of the responses generated. It highlights the complex non-linear relationships between shear force and deformation requiring the careful consideration of the applicable sensing range. Fabrication of the sensor design generated by the optimisation indicated how a simple change in the geometry from the original (Figure 1) led to a significant improvement in the sensitivity. This result could not be obtained prior to defining the mathematical formulations of the sensor response under load and the subsequent procedure developed for obtaining the optimal solution.

The designs which can be calculated from this work will continue to be fabricated as part of the ongoing prototype development of the MagOne sensor; this research will also include more precise manufacturing technologies. Additionally, the optimisation approach will be employed to investigate how a different selection of parameters in the design can be optimised. This could conceivably include design objectives relating to transient behaviour such as hysteresis or response time, both of which are of interest in the design of soft sensors. The numerical simulations would need to include additional models to describe this behaviour and the optimisation objectives adapted accordingly. The method developed in which the sensor characteristics are described by simulations and subsequent genetic programming derived metamodels is something which can be extended to a range of more complex applications and sensing modes. However, care will have to be taken when considering such problems because they will be subject to the curse of dimensionality as the number of variables becomes large and the problem becomes computationally inefficient to solve.

## 5. Conclusions

This paper demonstrates the complex non-linearity associated in determining the response of soft tactile sensors and subsequently how their designs can be optimised. This was achieved by incorporating numerical simulations to describe the sensor mechanics, metamodels to capture the sensor response over a range of variables, and optimisation to produce the best design. Experiments subsequently indicated that these designs were valid and an improvement on the original. It should be noted that there is a critical link between experimental testing and validation of the optimisation results in which such testing must be performed to ensure accuracy over the range of variables investigated. The methods introduced here will be used as part of developments in improving the performance of soft tactile sensors with the aim to substantiate this simulation and metamodel driven optimisation as a means of underpinning the design strategy in a range of tactile sensing problems.

## Figures and Tables

**Table 1:** Fixed design characteristics of the MagOne sensor.

Parameter	Value (Unit)
$H_{g}$	4.97 (mm)
$H_{m}$	2 (mm)
R	6 (mm)
$R_{h}$	1 (mm)
$R_{g}$	6 (mm)
$R_{m}$	2.5 (mm)
$\Delta_{z,\mathrm{sat}}$	2.97 (mm)
$\theta_{\max}$	0.42 (rad)

## Appendix A

This Appendix contains a flow chart of the optimisation procedure developed.

## References

[1] Bartolozzi C., Natale L., Nori F., Metta G. (2016). Robots with a sense of touch. Nat. Mater..

[2] Kappassov Z., Corrales J.A., Perdereau V. (2015). Tactile sensing in dexterous robot hands—Review. Robot. Auton. Syst..

[3] Dahiya R.S., Metta G., Valle M., Sandini G. (2010). Tactile sensing—From humans to humanoids. IEEE Trans. Robot..

[4] Lee H.K., Chung J., Chang S.I., Yoon E. (2011). Real-time measurement of the three-axis contact force distribution using a flexible capacitive polymer tactile sensor. J. Micromech. Microeng..

[5] Winstone B., Griffiths G., Melhuish C., Pipe T., Rossiter J. TACTIP–tactile fingertip device, challenges in reduction in size to ready for robot hand integration. Proceedings of the 2012 IEEE International Conference on Robotics and Biomimetics.

[6] Wettles N., Santos V.J., Johansson R.S., Loeb G.E. (2008). Biomimetic tactile sensor array. Adv. Robot..

[7] Clark J.J. A magnetic field based compliance matching sensor for high resolution, high compliance tactile sensing. Proceedings of the 1988 IEEE International Conference on Robotics and Automation.

[8] Jamone L., Natale L., Metta G., Sandini G. (2015). Highly sensitive soft tactile sensors for an anthropomorphic robotic hand. IEEE Sens. J..

[9] Wang H., de Boer G.N., Kow J., Alazmani A., Ghajari M., Hewson R.W., Culmer P.R. (2016). Design methodology for magnetic field based soft tri-axis tactile sensors. Sensors.

[10] Tomo T.P., Somlor S., Schmitz A., Jamone L., Huang W., Kristanto H., Sugano S. (2016). Design and characterization of a three-axis Hall effect based soft skin sensor. Sensors.

[11] De Oliveria T., Cretu A.M., Petriu E.M. (2017). Multimodal bio-inspired tactile sensing module. IEEE Sens. J..

[12] Wang H., de Boer G.N., Kow J., Alazmani A., Ghajari M., Hewson R.W., Culmer P.R. (2016). A low-cost soft tactile sensing array using 3D Hall sensors. Procedia Eng..

[13] Shin J., Spicer J.P., Abell J.A. (2012). Inverse and direct magnetic shaping problems. Struct. Multidiscip. Optim..

[14] Jia F., Liu Z., Zaitsev M., Henning J., Korvink J.G. (2014). Design multiple-layer gradient coils using least-squares finite element method. Struct. Multidiscip. Optim..

[15] Barthold F.J., Firuziaan M. (2000). Optimization of hyperelastic materials with isotropic damage. Struct. Multidiscip. Optim..

[16] Parsons R., Canfield S.L. (2002). Developing genetic programming techniques for the design of compliant mechanisms. Struct. Multidiscip. Optim..

[17] Lin J., Luo Z., Tong L. (2010). A new multi-objective programming scheme for topology optimization of compliant mechanisms. Struct. Multidiscip. Optim..

[18] Zhu X., Wang S. Development of soft sensor system via dynamic optimization. Proceedings of the 30th Annual Conference of the IEEE Industrial Electronics Society.

[19] Xu Z., Kolev S., Todorov E. Design, optimization, calibration, and a case study of a 3D-printed, low-cost fingertip sensor for robotic manipulation. Proceedings of the 2014 IEEE Conference on Robotics & Automation.

[20] Zheng Q.Z., Querin O.M., Barton D.C. (2006). Geometry and sizing optimisation of discrete structure using the genetic programming method. Struct. Multidiscip. Optim..

[21] Fawaz Z., Xu Y.G., Behdinan K. (2005). Hybrid evolutionary algorithm and application to structural optimization. Struct. Multidiscip. Optim..

[22] Maiolino F., Galantini F., Mastrogiovanni G., Gallone G., Cannata G., Capri F. (2015). Soft dielectrics for capacitive sensing in robot skins: Performance of different elastomer types. Sens. Actuators A Phys..

[23] Ribeiro P., Khan M.A., Alfadhel A., Kosel J., Franco F., Cardoso S., Bernardino A., Schmitz A., Santos-Victor J., Jamone L. (2017). Bio-inspired ciliary force sensor for robotic platforms. IEEE Robot. Autom. Lett..

[24] Nuelle K., Schultz M.J., Aden S., Dick A., Munske B., Gaa J., Kotlarski J., Ortmaier T. Force sensing, low-cost manipulator in mobile robotics. Proceedings of the 2017 IEEE Conference on Control, Automation and Robotics.

[25] Paulino T., Ribeiro P., Neto M., Cardoso S., Schmitz A., Santos-Victor J., Bernadino A., Jamone L. Low-cost 3-axis soft tactile sensor for the human-friendly robot Vizzy. Proceedings of the 2017 IEEE Conference on Control, Automation and Robotics.

[26] Wan L., Wang B., Wang Q., Han J., Cao S. (2017). The output characteristic of cantilever-like tactile sensor based on the inverse magnetoresistive effect. AIP Adv..

[27] Chung D.D.L. (2001). Electromagnetic interference shielding effectiveness of carbon materials. Carbon.

[28] Sparks J.L., Vavalle N.A., Kasting K.E., Long B., Tanaka M.L., Sanger P.A., Schnell K., Conner-Kerr T.A. (2015). Use of silicone materials to simulate tissue biomechanics as related to deep tissue injury. Adv. Skin Wound Care.

[29] Koza J. (1992). Genetic Programming: On the Programming of Computers by Means of Natural Selection.

[30] De Boer G.N., Wang H., Ghajari M., Alazmani A., Hewson R.W., Culmer P.R., Alboul L., Groß R., Melhuish C., Witkowski M., Prescott T.J., Penders J. (2016). Force and topography reconstruction using GP and MOR for the TACTIP soft sensor system. Towards Autonomous Robotic Systems TAROS 2016.

[31] COMSOL Inc., USA (2017). Comsol Multiphysics 5.3 [Computer Software]. https://www.comsol.com/.

[32] Dassault Systèmes, France (2016). Abaqus CAE 2017 [Computer Software]. https://www.3ds.com/.

[33] Zhang M., Mak A.F.T. (2009). In vivo frictional properties of human skin. Prosthet. Orthot. Int..

[34] Searson D. (2016). GPTIPS [Computer Software]. http://sites.google.com/site/GPTIPS4matlab/.

[35] The MathWorks Inc., USA (2017). Matlab R2017a [Computer Software]. https://www.mathworks.com/.

[36] Hazewinkel M. (2001). Minimax Principle, Encyclopaedia of Mathematics.

[37] Form2, USA (2017). Clear Resin GPCL02. https://www.formlabs.com/.

[38] Smooth-On Inc., USA (2017). EcoFlex 00-30. https://www.smooth-on.com/products/ecoflex-00-30/.

[39] Smooth-On Inc., USA (2017). Sil-poxy. https://www.smooth-on.com/product-line/sil-poxy/.

[40] ATI Industrial Automation, USA (2017). Nano17-E. https://www.ati-ia.com/.

[41] Zaber Technologies Inc., Canada (2017). T-LSR75B. https://www.zaber.com/.

[42] National Instruments, USA (2017). LabView 2017 [Computer Software]. https://www.ni.com/.

[43] Gupta G. (2012). Dynamic Low, High and Band Pass Filter [Computer Software]. https://uk.mathworks.com/matlabcentral/fileexchange/31985-dynamic-low--high-and-band-pass-filter/.

